# Determinants of the use of contraceptive methods by adolescents in the Democratic Republic of the Congo: results of a cross-sectional survey

**DOI:** 10.1186/s12905-022-02084-3

**Published:** 2022-11-28

**Authors:** Dieudonné M. Mpunga, Faustin M. Chenge, Thérèse NM. Mambu, Pierre Z. Akilimali, Mala A. Mapatano, Gilbert U. Wembodinga

**Affiliations:** 1 School of Public Health of Kinshasa, University of Kinshasa, Kinshasa 1, P.O.11850, Kinshasa, Democratic Republic of the Congo; 2 Centre de Connaissances Santé en RDC (CCSC), Kinshasa, Democratic Republic of the Congo; 3 School of Public Health of Lubumbashi, University of Lubumbashi, Lubumbashi, Democratic Republic of the Congo

**Keywords:** Determinants, Use, Contraception, Adolescent girls

## Abstract

**Background:**

Family planning (FP) is an effective strategy to prevent unintended pregnancies of adolescents. We aimed at identifying the socio-demographic factors underlying the low use of contraceptive methods by teenage girls in the Democratic Republic of the Congo (DRC).

**Methods:**

A secondary analysis targeting teenage girls aged 15–19 was carried out on the Performance, Monitoring and Accountability project 2020 (PMA 2020) round 7 data, collected in Kinshasa and Kongo Central provinces. The dependent variable was the “use of contraceptive methods by sexually active teenage girls”, calculated as the proportion of teenagers using modern, traditional or any contraceptive methods. Independent variables were: level of education, age, province, religion, marital status, number of children, knowledge of contraceptive methods and household income. Pearson's chi-square and logistic regression tests helped to measure the relationship between variables at the alpha significance cut point of 0.05.

**Results:**

A total of 943 teenagers were interviewed; of which 22.6, 18.1 and 19.9% ​​used any contraceptive method respectively in Kinshasa, Kongo Central and overall. The use of modern contraceptive methods was estimated at 9.9, 13.4 and 12.0% respectively in Kinshasa, Kongo Central and overall. However, the use of traditional methods estimated at 8.0% overall, was higher in Kinshasa (12.7%) and lower (4.7%) in Kongo Central (*p* < .001). Some factors such as poor knowledge of contraceptive methods (aOR = 8.868; 95% CI, 2.997–26.240; *p* < .001); belonging to low-income households (aOR = 1.797; 95% CI, 1.099–2.940; *p* = .020); and living in Kongo central (aOR = 3.170; 95% CI, 1.974–5.091; *p* < .001) made teenagers more likely not to use any contraceptive method.

**Conclusion:**

The progress in the use of contraceptive methods by adolescent girls is not yet sufficient in the DRC. Socio-demographic factors, such as living in rural areas, poor knowledge of FP, and low-income are preventing teenagers from using FP methods. These findings highlight the need to fight against such barriers; and to make contraceptive services available, accessible, and affordable for teenagers.

## Plain English summary

The use of contraceptive methods remains low among adolescents aged 15 to 19 in the Democratic Republic of the Congo. However, family planning (FP) methods can help to prevent unintended pregnancies. This study aimed at identifying the socio-demographic factors that prevent teenage girls from using FP methods. We analyzed the data from the Performance, Monitoring and Accountability project (PMA 2020), seventh round, collected in Kinshasa and Kongo Central provinces. The use of contraceptive methods by sexually active adolescents was measured according to the level of education, age, province, religion, marital status, number of children, knowledge of contraceptive methods and household income. For the 943 adolescent girls interviewed, the use of any contraceptive method was calculated at 22.6, 18.1 and 19.9%, respectively in Kinshasa, Kongo Central and overall. The use of traditional methods was estimated at 8.0% overall, higher in Kinshasa (12.7%) and lower (4.7%) in Kongo Central. However, the use of modern contraceptive methods was estimated at 9.9, 13.4 and 12.0% respectively in Kinshasa, Kongo Central and overall. Poor knowledge of contraceptive methods; low-income and living in Kongo central province were the factors associated with the low use of any contraceptive method. In conclusion, the progress in the use of contraceptive methods by adolescent girls is not yet sufficient, due to some socio-demographic barriers. These results suggest to fight against such factors; and to make contraceptive services available, accessible, and affordable for teenagers.

## Introduction

About half of pregnancies among adolescent women aged 15–19 living in developing countries are unintended; and more than half of these end in abortion, often under unsafe conditions [1]. Early pregnancies can have physical, emotional and socioeconomic impact in adolescent girls, families and the entire community [2, 3]. They often occur in low-income, low educated and dysfunctional families [4–7], in which alcohol consumption and early sexuality are common [8]. In the African region, teenage pregnancies are often followed by a drop out of schooling [7]; this fact is not only associated to poverty, but also makes some teenagers dependent on aid programs, and puts a considerable burden on economies and health systems [5, 6, 9].

The proportion of sexually active adolescents using contraceptive methods at age 19 is experiencing an annual increase wordwide, ranging from 2 to 17% in developing countries [10, 11]. However, the contraceptive prevalence remains low in teenagers. In 2019, 10.2% of adolescent girls were using any contraceptive method globally. In sub-Saharan Africa, the use of any contraceptive method was estimated at 10.1%, with large differences between married and unmarried adolescents [12]. Since 2006, unmet need for contraception has remained high (52%) among adolescent girls. During the same period, unmet needs in adults decreased from 45.8 to 38.0%, thus accentuating the inequalities between adults and adolescents [13]. In 12 countries, including the Democratic Republic of the Congo (DRC), a study in 2019 showed that the satisfaction of demand for modern contraceptive methods remained below 10% among the adolescent group [14]. Addressing unmet need would reduce unintended pregnancies by 6.0 million per year; which means avoiding 2.1 million unplanned births, 3.2 million abortions and 5600 maternal deaths [8]. Beyond improving the availability of contraceptive methods [15], a significant number of teenagers come up against obstacles, leading to contraceptive discontinuation and failure [10, 15, 16]. Obstacles, such as unfavorable legal and social environment [3]; limited contraceptive bargaining power; low knowledge of sexual and reproductive health (SRH) can result in risky sexual behaviors [3, 16]. The majority of teenagers use unreliable sources for information on SRH [17]; and out-of-school adolescents are more vulnerable and often make less informed choices [3]. Combining demand creation and the provision of information and user-friendly services can increase the uptake contraceptive methods [5, 18], by removing bottlenecks [19–21].

In the DRC, although the age of majority is set at 18 [22], more than half of adolescents become sexually active at age 17. In 2017, the use of contraceptive methods by single sexually active adolescent girls was estimated at 19.1 and 16.1%, respectively for modern and traditional methods. At the same moment, the contraceptive prevalence was 9.5% for modern methods and 6.3% for traditional methods among married or unionized adolescents. Unmet need for modern contraception was estimated at 56% among adolescent girls not in a relationship [23]. In 2016, up to six in 10 pregnancies that occurred in Kinshasa were unplanned; many of them ending in unsafe abortion [24]. To improve access to and uptake of family planning (FP) methods, some innovations, such as the community distribution of contraceptives [25–27] have been implemented. However, they appear to have little effect on the use of contraceptive methods by adolescents. The objective of this paper was to identify the socio-demographic determinants of the low use of contraceptive methods by adolescent girls in the DRC.

## Methods

In the DRC, the provision of FP services, including a range of contraceptive methods, are part of the minimum package of activities for all types of health facilities. Several approaches are used to offer contraceptive methods, including health facilities-based approaches, community-based distribution and social marketing strategy using private pharmacies. According to national Health System Strengthening Strategy (SRSS), the delivery of health care needs to be improved within health centers and hospitals.

### Study design

**Fig. 1:**
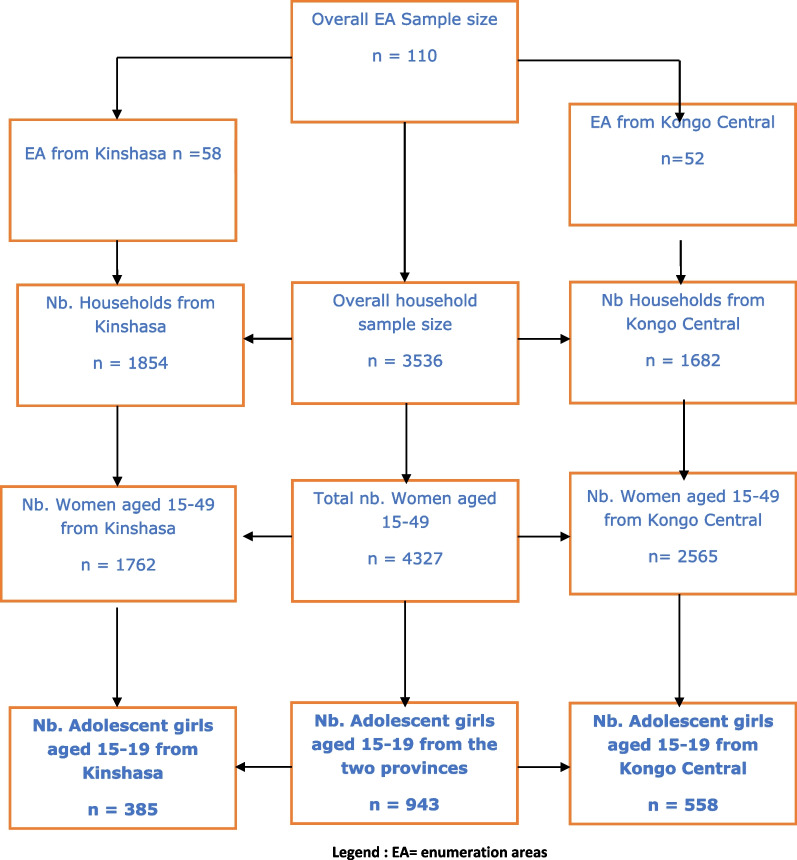
Study sampling procedure

A secondary analysis targeting adolescent girls aged 15–19 was carried out on the data of Round 7 of the Performance, Monitoring and Accountability project (PMA 2020) collected in 2018 [28]. The survey involved 3536 households, including 1854 in Kinshasa and 1682 in Kongo Central. A two-stage cluster design was used to select 110 enumeration areas (EA) in these provinces, using the selection probabilities proportional to size of the cluster. The sampling of EA and the enumeration of households were carried out before data collection. In each EA, some 33 households were selected by random sampling and all women aged 15–49 interviewed, after giving their informed consent. The sampling procedure is shown in Fig. 1.

### Dependent and independent variables

#### Dependent variable

Through this study, FP use was first measured as overall contraceptive use, then separated into modern and traditional methods (rhythm, withdrawal and other traditional methods such as folkloric methods like amulets, herbs, etc.). For specific methods, the following hierarchy was used to tabulate current use, selecting only the highest method in the list: female sterilization, male sterilization, implants (Norplant), intrauterine device (IUD), injectables, contraceptive pills, condoms, emergency contraception, standard day method (SDM), vaginal methods (foam, jelly, suppository), lactational amenorrhea method (LAM), periodic abstinence, withdrawal, and other methods. Current use of modern contraceptive methods was defined as use of sterilization, intrauterine device, injectables, implants, pills, standard day method using CycleBeads, male and female condoms, emergency contraception, lactational amenorrhea, and spermicides) [29].

The dependent variable was “use of contraceptive methods by sexually active teenage girls”, a dichotomus variable (1 = yes; 0 = no) calculated for any, modern and traditional contraceptive methods respectively. In the context of this work, sexually active adolescents are those who reported having had sex less than a year before the data collection.

#### Independent variables

The independent variables were chosen in the light of the current state of knowledge of the barriers to undertake FP services and methods.. Thus, a number of socio-demographic characteristics were examined including the province of residence (Kinshasa vs Kongo Central), age of adolescent girls (15–17 vs. 18–19), level of education (high vs low), marital status (married or cohabitating _in union vs. divorced, widowed, or never married_ not in union), religion, household income, knowledge of contraceptive methods, and number of children. The level of education was considered “high” if adolescents attended at least high school and above and low for adolescents who did not finish primary school. Several religions are practiced throughout the DRC. As part of this study, we first decided to group the adolescents into three groups: none, Christian and non-Christian. However, in order to make analysis, we grouped non-Christian and none as “non-Christian” vs. “Christian”, for adolescents practicing the Christian religion. Adolescent who spontaneously cited at least three FP methods were classified as having high knowledge of contraceptive methods; those who cited less than three FP methods were ranked as “poor knowledge of FP”. Household income (household wealth) was constructed using a wealth index based on ownership of 25 household assets, house material, livestock ownership and water source, which was converted into quintiles. According to the household income, two groups of adolescent girls were formed—one group as high income corresponding to households in the 3rd, 4th and 5th wealth quintile vs the other group as low income for households in the 1st and 2nd wealth quintile. According to the number of children, women were classified in two groups: one or more children vs none.

### Data collection and quality control

The data were collected by interviewers recruited from adult women living nearby and trained in data collection process, using mobile phones. They were previously validated, cleaned, weighted as part of the primary PMA study [28]. Analyzes were performed using Stata 14 software.

### Statistical analysis

Continuous variables were summarized as means or medians with their standard deviations or interquartile range as appropriate. The categorical variables were summarized as proportions with their confidence intervals. Bivariate analyzes were performed using Pearson's chi-square test to measure the association between dependent and independent variables taken individually. Logistic regression was performed by relating the dependent variable to independent variables pooled in a statistical model. All analyzes were performed using an alpha significance level of 0.05. Thus, all associations whose p-value was less than 0.05 were considered statistically significant.

### Ethics consideration

The PMA 2020 surveys received approval from the ethics committees of the Johns Hopkins School of Public Health and the Kinshasa School of Public Health (KSPH). The research protocol for performing secondary analyzes was reviewed and approved by the Ethics Committee of the KSPH under approval number ESP/CE/027/2018. All women aged 15–49 participating to the PMA study provided written and informed consent. The need for consent to participate from parent or legal guardian was waived, for young emancipated women aged 15–17, by the ethics committee of the KSPH (approval number ESP/CE/73B/2018). In the DRC, some of these adolescents are already married or living as a couple [30–32].

## Results

The household response rate was 95.3% for Kinshasa and 98.5% for Kongo Central. Interviews were completed with 943 adolescent girls aged 15–19. The average age of the interviewees was 16.97 ± 1.44 years old; most adolescents (58.3%) were between 15 and 17 years old. About 72.7% of respondents were in high school; 81.7% were single or never married; 66.4% lived with their parents and 84.7% had not yet given birth. Overall, about 51.2% of adolescent girls were sexually active, with the majority (62.4%) living in Kongo Central compared to 35.1% in Kinshasa (*p* < .001). The majority of teenagers (82.7%) were Christian, 14.2% not Christian and 3.1% did not practice any religion. About 24.9% of participants belonged to the highest economic quintile, while 15.0% came from the lowest economic quintile. Table 1

**Table 1:** Socio-demographic characteristics of adolescent girls interviewed in Kinshasa and Kongo Central, 2018 (n = 943)

Variables	Kinshasa (n = 385)	Kongo Central (n = 558)	Overall (n = 943)
*Age (yrs)*			
Mean ± SD	16.93 ± 1.41	16.99 ± 1.47	16.97 ± 1.44
15—17	232 (60.4)	319 (57.1)	551 (58.3)
18—19	152 (39.6)	240 (42.9)	392 (41.6)
*Household size*			
Mean ± SD	7.62 ± 3.11	6.06 ± 2.51	6.69 ± 2.87
*Education status*			
No formal education	1 (0.3)	31 (5.5)	32 (3.4)
Primary school	49 (12.8)	148 (26.5)	197 (20.9)
High school	310 (80.7)	376 (67.3)	686 (72.7)
University/Tertiary	24 (6.2)	4 (0.7)	28 (2.9)
*Sexual experience*			
No	250 (64.9)	210 (37.6)	460 (48.8)
Yes	135 (35.1)	348 (62.4)	483 (51.2)
*Marital status*			
Married/living in couple	34 (8.8)	107 (19.2)	141 (14.9)
Divorcee	0 (0.0)	10 (1.8)	10 (1.1)
Never married/Single	351 (91.2)	440 (79.0)	791 (84.0)
*Relationship with the head of household*			
Spouse	9 (2.3)	59 (10.6)	68 (7.2)
Daughter	231 (60.0)	395 (70.8)	626 (66.4)
Daughter-in-law	10 (2.6)	25 (4.5)	35 (3.7)
Granddaughter	64 (16.6)	32 (5.7)	96 (10.2)
Sister	29 (7.5)	10 (1.8)	39 (4.1)
Other	42 (10.9)	37 (6.6)	79 (8.4)
*Already given birth*			
No	356 (92.5)	440 (78.8)	796 (84.4)
Yes	29 (7.5)	118 (21.1)	147 (15.6)
*Number of children*			
0	359 (93.2)	449 (80.3)	808 (85.6)
1	23 (6.0)	95 (17.0)	118 (12.5)
2	3 (0.8)	15 (2.7)	18 (1.9)
*Religion*			
Christian	315 (81.8)	465 (83.3)	780 (82.7)
Non-Christian	61 (15.8)	73 (13.1)	134 (14.2)
None	9 (2.3)	20 (3.6)	29 (3.1)
*Wealth Quintile*			
Lowest	63 (16.4)	79 (14.1)	142 (15.0)
Lower	76 (19.7)	85 (15.2)	161 (17.1)
Middle	103 (26.7)	101 (18.1)	204 (21.6)
High	70 (18.2)	132 (23.6)	202 (21.4)
Highest	73 (18.9)	162 (29.0)	235 (24.9)

Based on unweighted data

An estimated number of 188 adolescent girls used any contraceptive method. The use of any contraceptive method was therefore estimated at 19.9% across the study, which represents 22.6% in Kinshasa and 18.1% in Kongo Central; with no significant difference between these provinces (*p* = .089).

However, modern contraceptive methods were more used than traditional methods. The use of modern contraceptive methods was estimated at 9.9, 13.4 and 12.0% respectively in Kinshasa, in Kongo Central and overall; and no significant difference was found between these provinces (*p* = .100). About 8.0% of adolescent girls used traditional contraceptive methods overall. However, in Kinshasa, the use of traditional contraceptive methods was estimated at 12.7%, higher than 4.7% reported in Kongo Central (*p* < .001). Overall, about 18.1% of adolescent girls had unmet needs for modern contraceptive methods; representing 11.2% in Kinshasa and 22.9% in Kongo Central. Unmet needs for FP were twice as high in Kongo Central compared to Kinshasa (*p* < .001). (Table 2).

**Table 2:** Use of contraceptive methods and unmet need for family planning among adolescent girls aged 15–19 in Kinshasa and Kongo Central, DRC, 2018 (n = 943)*

Variables	Overall (n = 943)	Kinshasa (n = 385)	Kongo Central (n = 558)	*P* value
*Use of all contraceptive methods (n, %)*				**.089**
Yes	188 (19.9)	87 (22.6)	101 (18.1)	
No	755 (80.1)	298 (77.4)	457 (81.9)	
*Use of modern contraceptive methods (n, %)*				**.100**
Yes	113 (12.0)	38 (9.9)	75 (13.4)	
No	830 (88.0)	347 (90.1)	483 (86.6)	
*Use of traditional contraceptive methods (n, %)*				< .001**
Yes	75 (8.0)	49 (12.7)	26 (4.7)	
No	868 (92.0)	336 (87.3)	532 (95.3)	
*Unmet need for family planning (n, %)*				< .001**
Yes	171 (18.1)	43 (11.2)	128 (22.9)	
No	772 (81.9)	342 (88.8)	430 (77.1)	

*The initial sample was weighted

**This probability reflects a statistically significant difference between the row and column variables

Six of 10 contraceptive methods used by adolescents were modern and four were traditional methods. Some adolescents used more than one contraceptive method. Thus, of all modern contraceptive methods, the foremost one used was the male condom (34.0%), followed by contraceptive pills (12.0%). The use of long-acting reversible contraceptive methods was estimated at 9.0% for implants and 1.0% for intra uterine devices (IUDs). Other modern contraceptive methods were poorly used; these are injectables (7%), emergency pill (5%), and cycle necklace (1%).

**Fig. 2:**
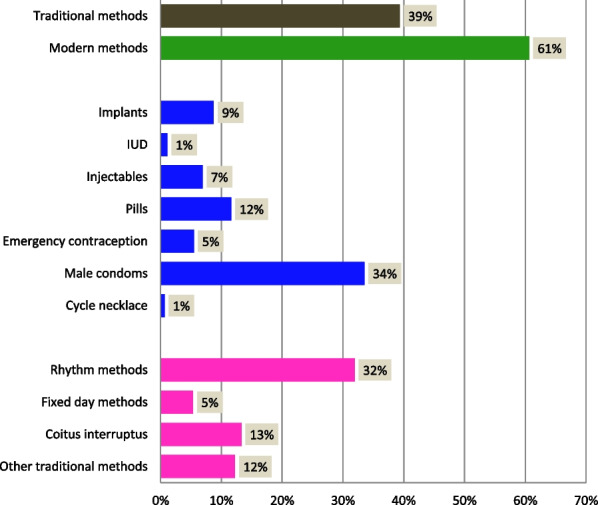
Contraceptive methods used by adolescent girls aged 15–19 in Kinshasa and Kongo Central, 2018 (n = 188)

Howerever, an important number of adolescent girls used traditional contraceptive methods as their first choice. Among these contraceptive methods, the most used were rhythm methods (32.0%); followed by coitus interruptus (13.0%) Fig. 2.

**Fig. 3:**
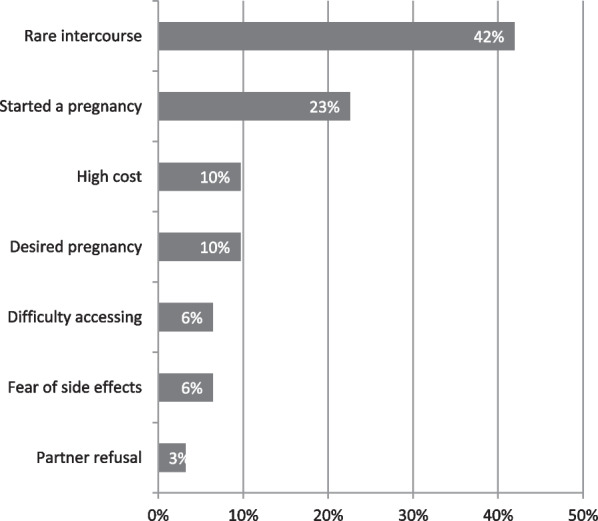
Reasons given by adolescents for cessation of contraceptive methods (n = 31)

Contraceptive methods used continuously over a long time are necessary to maintain the benefits in preventing unintended pregnancies. However, the data showed that 31 adolescent girls (16.5%) had stopped using FP methods at the time of the survey; mostly within the first year (data not shown); and due to several reasons. The most frequently mentioned reason was infrequent sex (42.0%), followed by early pregnancy (23.0%), high cost of contraceptives (10.0%) and desire to become pregnant (10.0%). A few numbers cited the accessibility of contraceptives methods (6.0%), side effects of contraceptives (6.0%) and partner refusal (3.0%) Fig. 3.

As for the sources of contraceptive methods for adolescents, the main source was pharmacies (44.0%), followed by health facilities (42.0%). Other sources, including community distributors, health providers and family members were poorly used (data not shown).

**Fig. 4:**
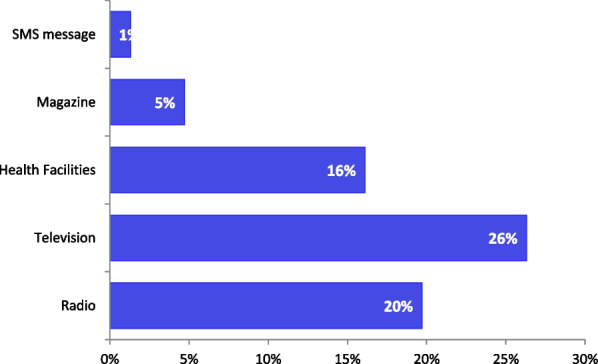
Sources of information on family planning for adolescent girls from Kinshasa and Kongo central (n = 943)

According to Fig. 4, several sources of information about FP were used by adolescent girls. However, of all, television was the most used by one in four (26%); followed by radio (20%) and health facilities (16%). The least used source of information was the short messaging (SMS) (1%) Fig. 4.

**Fig. 5:**
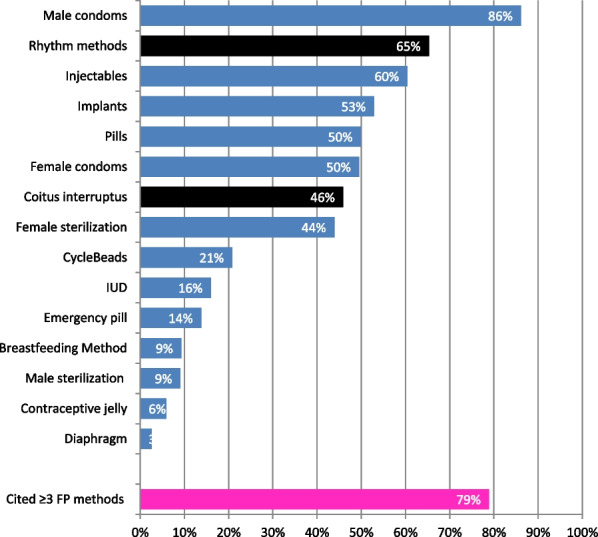
Contraceptive methods spontaneously cited by adolescent girls from Kinshasa and Kongo Central (n = 943)

Teenagers spontaneously mentioned fifteen contraceptive methods they had already heard of. Of these, 13 (86.7%) were modern methods and two (13.3%) were traditional methods. The best-known FP methods were the male condom (86.0%), rhythm methods (65.0%), injectables (60.0%), implants (53.0%), contraceptive pills (50.0%), female condom (50.0%), coitus interruptus (46.0%) and female sterilization (44.0%). The least known FP methods included diaphragm (3.0%), contraceptive jelly (6.0%), male sterilization (9.0%), breastfeeding method (9.0%), emergency pill (14.0%), IUDs (16.0%) and cyclebeads (21.0%). As part of the assessment of adolescents' knowledge of contraceptive methods, we checked how many methods they had heard about. Thus, 79.0% adolescent girls cited at least 3 out of 15 contraceptive methods. A few adolescents (21.0%) cited less than 3 contraceptive methods among those they had heard of Fig. 5.

The use of modern or traditional contraceptive methods was influenced by some characteristics of adolescents. We present the results of the bivariate analysis using the Pearson Chi-square test. According to these results, on one hand, the use of traditional contraceptive methods by sexually active adolescent girls was associated with five variables, including knowledge of contraceptive methods (*p* = .002), level of education (*p* = .022), religion (*p* = .007), number of children (*p* = .046) and province (*p* < .001). On the other hand, the use of modern contraceptive methods was associated with two variables, including knowledge of contraceptive methods (*p* < .001) and household income (*p* = .002). However, six independent variables were associated with the use of any contraceptive method by sexually active adolescents. These are knowledge of contraceptive methods (*p* < .001), level of education (*p* = .002), religion (*p* = .017), number of children (*p* = .039), household income (*p* = .008) and province (*p* < .001). As shown in Table 3, only knowledge of contraceptive methods was associated with the use of any, modern and traditional FP methods. Table 3

**Table 3:** Use of contraceptive methods according to socio-demographic characteristics of adolescent girls aged 15–19 from Kinshasa and Kongo Central provinces, DRC (n = 943)**

Variables	Any contraceptive methods			Modern contraceptive methods			Traditional contraceptive methods		
	No (n, %)	Yes (n, %)	*P* value	No (n, %)	Yes (n, %)	P value	No (n, %)	Yes (n, %)	*P* value
Age Group (years)			**.395**			**.109**			**.438**
15–17	129 (42.0)	67 (38.1)		159 (42.5)	37 (33.9)		165 (39.8)	30 (44.8)	
18–19	178 (58.0)	109 (61.9)		215 (57.5)	72 (66.1)		250 (60.2)	37 (55.2)	
Knowledge of FP methods			< .001^*^			< .001^*^			.002^*^
Poor	80 (26.1)	4 (2.3)		83 (22.1)	1 (0.9)		81 (19.5)	3 (4.4)	
High	227 (73.9)	172 (97.7)		292 (77.9)	107 (99.1)		334 (80.5)	65 (95.6)	
Education status			.002^*^			**.113**			.022^*^
Low	105 (34.2)	37 (21.0)		116 (31.0)	25 (23.1)		130 (31.3)	12 (17.6)	
High	202 (65.8)	139 (79.0)		258 (69.0)	83 (76.9)		286 (68.8)	56 (82.4)	
Marital status			**.186**			**.930**			**.087**
Single / widowed / divorced	203 (66.3)	127 (72.2)		255 (68.4)	75 (68.8)		278 (67.1)	52 (77.6)	
Married/ living as a couple	103 (33.7)	49 (27.8)		118 (31.6)	34 (31.2)		136 (32.9)	15 (22.4)	
Religion			.017^*^			**.527**			.007^*^
Christian	259 (84.4)	133 (75.6)		306 (81.6)	86 (78.9)		345 (82.9)	47 (69.1)	
Non- Christian	48 (15.6)	43 (24.4)		69 (18.4)	23 (21.1)		71 (17.1)	21 (30.9)	
Already given birth			.039^*^			**.462**			.046^*^
No	218 (71.0)	140 (79.5)		275 (73.3)	83 (76.9)		301 (72.4)	57 (83.8)	
Yes	89 (29.0)	36 (20.5)		100 (26.7)	25 (23.7)		115 (27.6)	11 (16.2)	
Household income			.008^*^			.002^*^			**.938**
Low	128 (41.7)	52 (29.5)		153 (40.8)	27 (24.8)		155 (37.3)	25 (36.8)	
High	179 (58.3)	124 (70.5)		222 (59.2)	82 (75.2)		261 (62.7)	43 (63.2)	
Provinces			< .001^*^			**.065**			< .001^*^
Kongo Central	253 (82.4)	95 (54.0)		278 (74.1)	71 (65.1)		324 (77.9)	25 (36.8)	
Kinshasa	54 (17.6)	81 (46.0)		97 (25.9)	38 (34.9)		92 (22.1)	43 (63.2)	

**Based on weighted data

*This probability reflects a statistically significant difference between the row and column variables

The logistic regression (Tables 4, 5, 6) helped to measure the relationship between the “use of any, modern or traditional contraceptive methods by sexually active adolescent girls” and the independent variables. According to the results in Table 4, before and after adjusting the data, the use of modern contraceptive methods by sexually active teenagers was associated to adolescent’s knowledge of contraceptive methods (*p* = .002). Teenagers with poor knowledge of FP were significantly more likely not to use modern contraceptive methods compared to the second group (Adjusted OR = 23.100; 95%CI, 3.295–161.942; *p* = .002). Table 4

**Table 4:** Factors associated with the low use of modern contraceptive methods by adolescent girls aged 15–19 from two provinces of the DRC (n = 483)

Variables	Nb./n (%)	Crude OR (95% CI)	*P* value	Adjusted OR (95% CI)	*P* value
*Knowledge of FP methods*					
Poor	83/84 (98.8)	18.711 (2.473–141.591)	.005*	23.100 (3.295–161.942)	.002*
High	292/399 (73.2)	–		–	
*Educational status*					
Low	116/141 (82.3)	0.825 (0.440–1.564)	.548	0.940 (0.533–1.659)	.832
High	258/341 (75.6)	–		–	
*Age groups (years)*					
15–17	159/196 (81.1)	1.563 (0.957–2.553)	.074	1.533 (0.942–2.496)	.085
18–19	215/287 (74.9)	–		–	
*Marital Status*					
Never married/Single	255/330 (77.3)	0.746 (0.409–1.361)	.340	1.015 (0.597–1.725)	.957
Married/living in couple	118/152 (77.6)	–		–	
*Religion*					
Christian	306/392 (78.1)	0.958 (0.533–1.722)	.887	1.141 (0.644–2.020)	.651
Non-Christian	69/92 (75.0)	–		–	
*Number of children*					
None	275/358 (76.8)	0.788 (0.430–1.443)	.441	0.754 (0.418–1.359)	.347
At least one	100/125 (80.0)	–		–	
*Household income*					
Low	153/180 (85.0)	1.472 (0.838–2.584)	.102	1.625 (0.941–2.807)	.081
High	222/304 (73.0)	–		–	
*Provinces*					
Kongo Central	278/349 (79.7)	0.864 (0.525–1.420)	.563	1.181 (0.711–1.962)	.521
Kinshasa	97/135 (71.9)	–		–	

*This probability reflects a statistically significant difference between independent and dependent variables

**Table 5:** Factors associated with the low use of traditional contraceptive methods by adolescent girls aged 15–19 from two provinces of the DRC (n = 483)

Variables	Nb./n (%)	Crude OR (95% CI)	*P* value	Adjusted OR (95% CI)	*P* value
*Knowledge of FP methods*					
Poor	81/84 (96.4)	2.552 (0.561–11.596)	.225	2.216 (0.592–8.293)	.237
High	334/399 (83.7)	–		–	
*Educational status*					
Low	130/142 (91.5)	1.779 (0.787–4.020)	.166	1.484 (0.703–3.135)	.301
High	286/342 (83.6)	–		–	
*Age groups (years)*					
15–17	165/195 (84.6)	0.736 (0.423–1.285)	.280	0.761 (0.421–1.376)	.367
18–19	250/287 (87.1)	–		–	
*Marital Status*					
Never married/Single	278/330 (84.2)	0.644 (0.302–1.373)	.254	0.744 (0.370–1.500)	.409
Married/living in couple	136/151 (90.1)	–		–	
*Religion*					
Christian	345/392 (88.0)	2.060 (1.103–3.848)	.023*	2.009 (1.039–3.885)	.038*
Non-Christian	71/92 (77.2)	–		–	
*Number of children*					
None	301/358 (84.1)	0.663 (0.303–1.453)	.305	0.875 (0.395–1.937)	.741
At least one	115/126 (91.3)	–		–	
*Household income*					
Low	155/180 (86.1)	1.068 (0.588–1.941)	.828	1.396 (0.740–2.631)	.303
High	261/304 (85.9)	–		–	
*Provinces*					
Kongo Central	324/349 (92.8)	4.157 (2.254–7.665)	< .001*	5.372 (2.904–9.637)	< .001**
Kinshasa	92/135 (68.1)	–		–	

*This probability reflects a statistically significant difference between independent and dependent variables

**Table 6:** Factors associated with the low use of any contraceptive methods by adolescent girls aged 15–19 from two provinces of the DRC (n = 483)

Variables	Nb./n (%)	Crude OR (95% CI)	*P* value	Adjusted OR (95% CI)	*P* value
*Knowledge of FP methods*					
Poor	80/84 (95.2)	9.895 (2.892–33.857)	< .001*	8.868 (2.997–26.240)	< .001*
High	227/399 (56.9)	–		–	
*Educational status*					
Low	105/142 (73.9)	1.137 (0.634–2.038)	.667	1.122 (0.668–1.883)	.664
High	202/341 (59.2)	–		–	
*Age groups (years)*					
15–17	129/196 (65.8)	1.200 (0.766–1.882)	.426	1.244 (0.803–1.929)	.328
18–19	178/287 (62.0)	–		–	
*Marital Status*					
Never married/Single	203/330 (61.5)	0.637 (0.370–1.097)	.104	0.883 (0.541–1.441)	.620
Married/living in couple	103/152 (67.8)	–		–	
*Religion*					
Christian	259/392 (66.1)	1.587 (0.929–2.712)	.091	1.650 (0.981–2.774)	.059
Non-Christian	48/91 (52.7)	–		–	
*Number of children*					
None	218/358 (60.9)	0.668 (0.384–1.163)	.154	0.754 (0.441–1.289)	.302
At least one	89/125 (71.2)	–		–	
*Household income*					
Low	128/180 (71.1)	1.454 (0.874–2.418)	.149	1.797 (1.099–2.940)	.020*
High	179/303 (59.1)	–		–	
*Provinces*					
Kongo Central	253/348 (72.7)	2.195 (1.377–3.498)	.001*	3.170 (1.974–5.091)	< .001*
Kinshasa	54/135 (40.0)	–		–	

*This probability reflects a statistically significant difference between independent and dependent variables

As shown in Table 5, before and after adjusting the data, two independent variables were significantly associated with the “use of traditional FP methods by sexually active adolescent girls”. These variables are religion (*p* < .038) and the province of residence (*p* < .001). On one hand, Christian teenagers were significantly more likely not to use traditional FP methods than non-Christian adolescents (Adjusted OR = 2.009; 95%CI, 1.039–3.885; *p* = .038). On the other hand, adolescent women living in Kongo Central were significantly more likely not to use traditional contraceptive methods than those living in Kinshasa (Adjusted OR = 5.372; 95%CI, 2.904–9.637; *p* < .001). Table 5

Unlike Tables 4 and 5, the results of Table 6 show that 3 independent variables were associated with the use of any contraceptive method by sexually active adolescent girls; these are knowledge of contraceptive methods, household income and province. Thus, characteristics such as low knowledge of contraceptive methods (Adjusted OR = 8.868; 95%CI, 2.997–26.240; *p* < .001); belonging to low-income households (Adjusted OR = 1.797; 95%CI, 1.099–2.940; *p* = .020); and living in Kongo Central province (Adjusted OR = 3.170; 95%CI, 1.974–5.091; *p* < .001) made teenagers more likely not to use any contraceptive methods than those without these factors. Table 6

## Discussions

The results of this study confirm the upward trend in contraceptive prevalence provided through the last Multiple Indicator Cluster Survey (MICS-RDC 2018) [23]. When compared to the results of the last Demographic and Health Survey (DHS-RDC 2013–14) [33]; and the targets set through the multisector FP strategic plan [34], these findings suggest an improvement in the use of contraceptive methods, particularly among never-married adolescents. In 2014, the DHS-RDC reported a contraceptive prevalence of 11.5, 5 and 6.5% in adolescents; respectively for any, modern and traditional FP methods [33]. These results are consistent with what have been reported in the literature. Never-married women usually have the highest contraceptive prevalence than currently married women [35].

Although clearly increasing, the proportion of adolescent girls using contraception in the DRC remains low compared to the African average. Actually, from 2013 to 2019, the use of modern contraceptive methods improved less among sexually active adolescents (married or living as a couple), from 5.4 to 9.5% than in adult women, among whom it ranked from 7.8 to 17.6% [23, 33]. However, these results contrast with those found by Kennedy et al. [36] and Munakampe et al. [17] who reported more progress in adolescents’ contraceptive use compared to women aged 20–49.

Our results indicated a significant decrease in unmet FP needs among adolescents compared with previous studies, namely DHS-RDC 2013 [33] and MICS-RDC, 2018 [23]. This obsevation can be explained by the fact that the aforementioned studies were organized at the national level; while this study took place only in Kinshasa and Kongo Central. These provinces located near the political decision-makers have benefited significant support from the Ministry of Health (MOH). However, according to the findings of this study, adolescent girls residing in the rural area of Kongo Central have higher unmet needs for contraception than those living in the urban area (Kinshasa). These results suggest a problem of equity in the distribution of FP services between rural and urban areas, already highlighted by Mpunga et al*.* [20]. Although contraceptive prevalence is high among women in Kongo Central, compared to that of Kinshasa, Kongo Central also registers a significant proportion of unmet need. This paradoxical result can be explained by the results in Table 1 which showed that the proportion of women in union is high in Kongo Central compared to Kinshasa. A previous study has shown that never-married women are significantly more likely to have their demand for contraceptive use satisfied than formerly married women [35]. According to Gilda et al. [37], women with unmet needs for FP cite infrequent sex, unmarried status, and the side effects of contraceptive methods as discouraging the use of modern contraception. These observations are consistent with the results in Fig. 3.

This weak progress nevertheless reflects the considerable efforts made by the government of the DRC with a gradual improvement of the supply of FP methods [25] and the development of innovative approaches [26, 27].

Given to these results, the DRC could not reach the target of the FP strategic plan set at least 19% of modern contraceptive prevalence by 2020 [34]. Barriers to accessing contraceptive services, related to supply and demand; and misconceptions of providers, users and the community about some modern methods could explain this state of affairs [38]. Other barriers are related to the health system, including the low availability of user-friendly services [20, 21] and the issue of equity [39]. Health facilities (HFs) are often inaccessible, unacceptable, inappropriate and ineffective for disadvantaged adolescents [40]. Some HFs not offering traditional methods are likely not to be used by some adolescents [21].

Other barriers are related to the characteristics of adolescent girls. We showed that the practice of the Christian religion could hinder the use of certain contraceptive methods by adolescent girls. However, some adolescent girls are not attached to Christian values that encourage the practice of sexuality only within the framework of marriage. One study showed that unmarried teens who regularly attend religious services are less likely to have ever had sex than others [41]. It is shown that most sexually active women of all beliefs—including Catholic women—whether single or married, use contraception when they do not want to become pregnant [41]. Few studies have assessed the relationship between adolescent religious attendance and contraceptive use, and their results are mixed. Consistent with our findings, some studies have found that adolescents with higher religiosity or more conservative religious affiliations are less likely than others to use birth control methods during sex [42, 43]. Other researchers have found no association between religion and contraceptive uptake [44, 45].

Limited knowledge about contraception has been shown to be a major barrier to accessing and using this service, especially among unmarried adolescents [17, 46–48]. Considering the results of this work, we found that the majority of adolescents cited at least three different contraceptive methods. Despite this fact, only one in five teenage girls uses any contraceptive method. Thus, this result suggests organizing a more in-depth analysis of the unfavorable attitudes to contraception which could be linked to negative cultural attitudes, in particular towards premarital sex [49]. According to Sneha et al. [50], adolescent girls' willingness and ability to use contraceptive services are often negatively affected by interpersonal influences (peers, partners, and parents); community influences (social norms) and macro-social influences involving religion in particular. Munakampe et al. [17] and Borraccino et al. [51] suggest the implementation of interventions involving parents and teachers in order to convey healthy messages to adolescents. Parental control discriminates more against the sexual behavior of adolescents [52], open parental communication on sexuality and comprehensive sexual education at school appear to be protective factors against the occurrence of early and unplanned pregnancies. These factors identified by Krugu et al. [53] should be targeted by intervention programs at the individual, interpersonal, school and community levels. Apart from sex education by parents, the attitude of health providers needs to be improved to facilitate the use of contraceptive services [54, 55].

Male condoms are the best known and most widely used contraceptive method among teenage girls, followed by rhythm methods. These results corroborate those found during the 2013 DHS survey [33]. The effectiveness of most natural/traditional methods is high, varying from 90 to 98% if the method is understood and well used by the couple [56, 57]; coitus interruptus being the least effective method. In this study, we have shown that four out of ten adolescent girls use traditional contraceptive methods, mainly in Kinshasa compared to Kongo Central. This observation could be explained by the level of education which is usually high in urban areas unlike in rural areas. However, most traditional FP methods depend on user intervention and then have a greater risk of failure than those that do not depend on the user. The success of natural methods largely depends on the level of education, knowledge and practices of the users [58]. Adolescents must therefore benefit from effective sex education programmes; to achieve this goal, guidelines on sex education can be applied [59].

## Strengths and limitations

Limitations of our analysis include the fact that the data on other determinants of contraceptive use, access to health information and services, discussion between couples, women’s autonomy, and cultural barriers were not available. Dichotomizing covariates in this study can lead to residual counfounding. Other weakness lies in the fact that the study took place in two provinces of the DRC, not nationwide representation. Due to the small number of observations concerning certain variables, some confidence intervals are wide. Finally, because the analyses used cross-sectional data, causality could not be determined. However, this study covered a representative sample of adolescent girls aged 15–19 whose selection was made randomly. The data were weighted as part of the analyses.

## Conclusions

This study shows an early improvement in the uptake of contraceptive methods among adolescent girls aged 15–19 in the DRC. However, the progress in the use of FP methods is not yet sufficient. A significant proportion of adolescents use traditional contraceptive methods; and others are not yet using any contraceptive method. Unmet FP needs are gradually decreasing, but remain high, especially in rural areas. The use of contraceptive methods by adolescent girls is influenced by some socio-demographic charasteristics. Poor knowledge of contraceptive methods, low household income as well as residence in rural areas prevent adolescents from using any contraceptive methods. In order to improve the use of contraception, in addition to actions on socio-demographic factors, it is necessary to make contraceptive services available and accessible for adolescents. Comprehensive sexuality education programs should be offered by schools, health providers and parents.

## Data Availability

The datasets used in this study are the data of the PMA 2020 Round 7, 2018. They can be obtained on reasonable request to be sent to the PMA team.

## Abbreviations

FP: Family planning
DRC: Democratic Republic of the Congo
SRH: Sexual and reproductive health
EA: Enumeration areas
PMA 2020: Performance Monitoring Accountability 2020
IUDs: Intrauterine devices
MICS: Multi indicators clusters suvery
DHS: Demographic and health survey
MOH: Ministry of Health
RHS: Reproductive health service
RIPSEC: Renforcement Institutionnel pour des Politiques de Santé basées sur l’Evidence au Congo
HFs: Health facilities
WHO: World Health Organization
ESPK: Ecole de Santé Publique de Kinshasa
